# A case report of acute cyanide poisoning treated with lactate as an indicator

**DOI:** 10.1097/MD.0000000000045896

**Published:** 2025-11-07

**Authors:** Mitsuko Suzuki, Saki Takeda, Kazuki Sugaya, Masahiro Iwabuchi, Rie Zenda, Tsuyoshi Suzuki, Makoto Onodera, Ken Iseki

**Affiliations:** a Department of Regional Emergency Medicine, Fukushima Medical University, Fukushima, Japan; b Department of Forensic Medicine, Fukushima Medical University, School of Medicine, Fukushima, Japan; c Department of Emergency and Critical Care Medicine, Fukushima Medical University, School of Medicine, Fukushima, Japan; d Department of Disaster Medicine, Fukushima Medical University Hospital, Fukushima, Japan; e Department of Radiation Disaster Medicine, Fukushima Medical University, School of Medicine, Fukushima, Japan.

**Keywords:** cyanide poisoning, lactate, lactic acidosis

## Abstract

**Rationale:**

Cyanide poisoning is a life-threatening condition that impairs cellular oxygen utilization, leading to lactic acidosis. However, serum cyanide levels are not readily available in clinical settings, making diagnosis and treatment monitoring difficult. In this report, we describe the treatment of a patient with severe cyanide poisoning using serum lactate levels as an indicator of the effectiveness of the treatment.

**Patient concerns:**

A 70-year-old man with suspected cyanide poisoning was transported to the emergency department in an unconscious state approximately 45 minutes after poisoning.

**Diagnoses:**

Laboratory findings revealed severe lactic acidosis (pH 7.07) with a high lactate level (19.0 mmol/L) and hypertension. Based on his history and clinical presentation, cyanide poisoning was strongly suspected.

**Interventions:**

The patient received antidotes and supportive care, including high-concentration oxygen therapy and antihypertensive medication. Serum lactate levels and blood cyanide concentrations were periodically measured to monitor treatment response.

**Outcomes:**

The patient’s consciousness improved with decreasing lactate levels, achieving full consciousness approximately 9 hours after poisoning. Finally, he was discharged from the intensive care unit without apparent sequelae and transferred to a psychiatric ward.

**Lessons:**

This case highlights the utility of serum lactate as an accessible biomarker for diagnosing cyanide poisoning and evaluating treatment efficacy. Given the unavailability of rapid cyanide assays in most clinical settings, serial lactate monitoring may aid in guiding therapeutic decisions.

## 1. Introduction

Cyanide compounds are toxic substances accessible to the general public owing to their use in industries, such as precious metal production and car body repair. Cyanide poisoning has a high mortality rate owing to its rapid post-exposure effects, impairing the utilization of cellular oxygen. Once ingested, cyanide compounds are rapidly absorbed in the stomach as hydrogen cyanide and exert toxicity through cyanide ions.^[1]^ These ions bind to cellular cytochrome oxidase and interfere with aerobic metabolism, which generates intracellular adenosine triphosphate.^[2]^ As intracellular adenosine triphosphate depletes, anaerobic metabolism accelerates, leading to increased lactic acid production and venous blood oxygen saturation.^[3]^ Given the high oxygen demand of the brain and heart, early symptoms primarily affect the central nervous and cardiovascular systems. In severe cases, poisoning can result in coma, convulsions, circulatory failure, and death.^[3]^ Diagnosing cyanide poisoning or determining the effectiveness of treatment in clinical settings is challenging, as direct blood cyanide measurement is often impractical. Therefore, in this case, we used lactate levels with blood gas analysis as an indicator of treatment effectiveness, measuring them periodically along with blood cyanide concentrations in collected samples.

## 2. Case presentation

**Figure 1.**
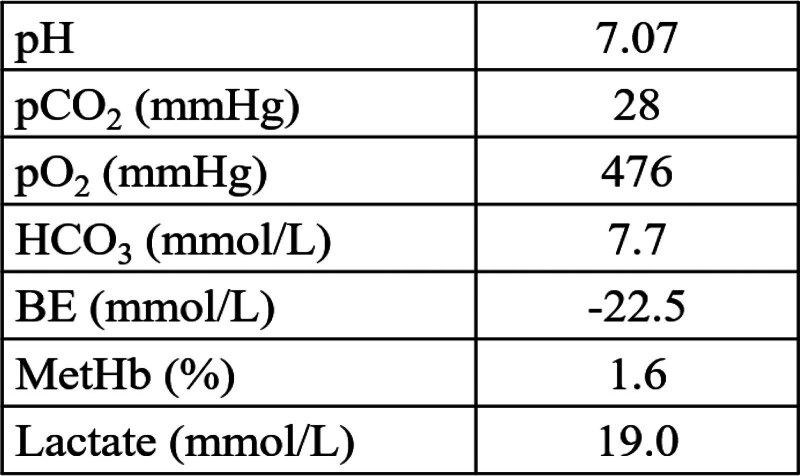
Shows the first sequence of arterial blood gases under 10 L O_2_ administered at arrival in emergency depart. This is a finding indicative of marked lactic acidosis.

**Figure 2.**
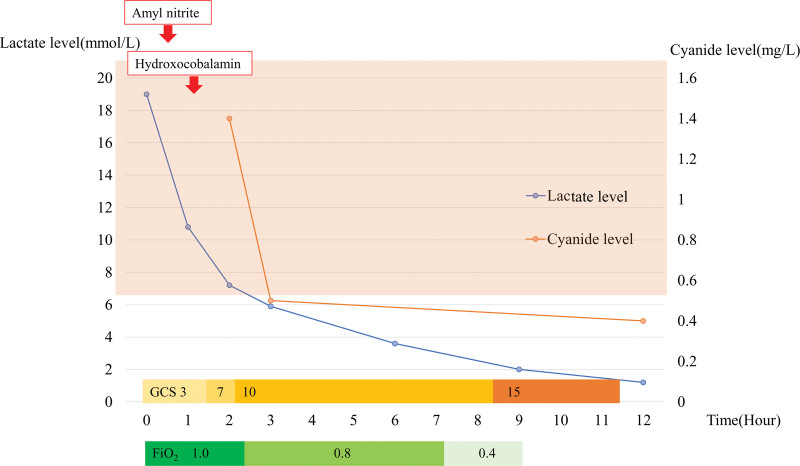
Shows the lactate level and cyanide level measurement plotted against time after visit. Lactate levels and cyanide levels decreased alongside consciousness levels after antidote administration, and oxygen concentration was reduced accordingly.

A 70-year-old man with a history of hypertension and diabetes ingested powder from a container labeled “potassium cyanide” following a dispute with his family. Immediately after ingestion, he became unstable and collapsed. Emergency medical services arrived approximately 17 minutes post-ingestion and found him in a severely comatose state with a Glasgow Coma Scale (GCS) score of E1V1M1. Upon presentation, his vital signs included a pulse rate of 99 beats/min, blood pressure of 184/110 mm Hg, a respiratory rate of 18 breaths/min, and an oxygen saturation of 100% with 10 L of oxygen administered. Based on his history, cyanide poisoning was suspected, and the emergency medical service transported him to the emergency department approximately 45 min after the poisoning while communicating this information. On physical examination, the patient had a GCS score of 3 with sluggish light reflexes. No carotid-jugular distention, thoracic movement disorder, peripheral cold sensation, or livedo reticularis of the anterior chest was observed. His jaw was rigid, limiting mouth opening. Excessive oral secretions and tongue retraction were observed; however, a typical bitter almond odor was not detected. Arterial blood gas analysis revealed severe lactic acidosis (pH 7.07) with a lactate level of 19 mmol/L (Fig. 1). The urine drug screen test (IVeX-screen®, BioDesign Co., Ltd., Tokyo, Japan) was negative. Chest X-ray and computed tomography revealed no acute pathology. A 12-lead electrocardiogram was performed upon arrival, revealing no abnormalities. As his blood pressure remained high, calcium channel blockers were administered. He was intubated with rocuronium and fentanyl and administered 0.25 mL of amyl nitrite inhalation every 5 minutes. After 2 doses, he received 5 g of hydroxocobalamin (Cyanokit®, Merck Biopharma Co., Ltd., Tokyo, Japan) and was admitted to the intensive care unit (ICU). After admission, we began monitoring his arterial pressure and electrocardiogram. Ventricular bigeminy was observed immediately after ICU admission; therefore, intravenous lidocaine (10 mg) was administered, after which the ECG reverted to sinus rhythm. No further arrhythmias were observed thereafter. Lactate levels and blood cyanide concentrations were monitored periodically. Blood samples were analyzed using headspace gas chromatography with a nitrogen-phosphorus detector to determine blood cyanide concentrations. Changes in lactate levels and blood cyanide concentrations are presented in Figure 2. The lactate and consciousness levels improved accordingly. Oxygen therapy was initially initiated at 100% concentration and gradually reduced following improvement in lactate levels. His blood pressure remained relatively stable after admission to the ICU. Nine hours after the poisoning, lactate levels decreased to 2.0 mmol/L, and his GCS score improved to E4VTM6. He was extubated and could speak normally. After extubation, the patient required higher doses of antihypertensive medication, possibly owing to the discontinuation of analgesics. On hospital day 3, he was transferred to the psychosomatic medicine department for detailed examination and treatment of psychiatric disorders. On day 29, cognitive assessment using the Revised Hasegawa’s Dementia Scale,^[4]^ which is widely used as a screening tool for dementia in Asian countries, yielded a score of 28 (maximum: 30), indicating no significant brain dysfunction.

## 3. Discussion

Cyanide poisoning disrupts aerobic metabolism, leading to severe lactic acidosis. As serum cyanide levels cannot be rapidly measured in clinical settings, diagnosing cyanide poisoning and assessing treatment efficacy can be challenging. A significant increase in lactate production can be used to diagnose cyanide poisoning. Baud et al^[5]^ studied the relationship between lactate levels and blood cyanide concentrations in patients with cyanide poisoning and suggested that patients with high blood cyanide levels (>1.0 mg/L) also had high lactate levels (>8 mmol/L). Therefore, clinicians should suspect cyanide poisoning in cases of unexplained hyperlactatemia, particularly in patients with symptoms such as respiratory failure, abnormal pulse rate, and even coma. In our case, cyanide poisoning was suspected based on the clinical history obtained by the emergency services. Severe lactic acidosis on arrival supported the diagnosis, and the severely comatose state immediately after poisoning suggested that the ingested doses may have been lethal. Generally, a blood cyanide level of 0.5 to 1.0 mg/L indicates toxicity, whereas a level of 2.5 to 3.0 mg/L is considered lethal.^[6]^ The half-life of cyanide in the blood is estimated to be approximately 1 hour.^[7]^ In our case, at 3 hours after poisoning, the blood cyanide level was 1.4 mg/L (Fig. 2), suggesting a potentially lethal dose immediately after poisoning.

Treatment for cyanide poisoning involves early administration of antidotes^[2,3]^ and supportive therapy, such as high-concentration oxygen administration and circulatory management. Hydroxocobalamin is administered at an initial dose of 5 g, with additional doses of 5 g possible as needed.^[3]^ However, as amyl nitrite and sodium nitrite induce methemoglobin, care must be taken to avoid excessive methemoglobin levels when administering these agents.^[3]^ The package insert recommends that antidotes for cyanide poisoning be administered “without delay.” Supporting this, a retrospective study investigating the time to hydroxocobalamin administration demonstrated that the interval from hospital arrival to antidote administration was significantly longer in non-survivors than in survivors.^[8]^ In the present case, the patient was transported to the emergency department 45 minutes after the poisoning, and the emergency medical services had already communicated the suspicion of cyanide poisoning prior to arrival. This early recognition allowed for the prompt initiation of supportive therapy and antidote administration, which was likely a key factor contributing to the survival of the patient. Cyanide poisoning can cause adverse effects on all organs, owing to cellular asphyxia. Therefore, supportive therapy for cyanide poisoning often requires respiratory support and circulatory management. In terms of circulation, cyanide poisoning initially presents with hypertension, which is typically followed by hypotension as a manifestation of cardiovascular suppression, often necessitating circulatory support.^[1]^ On the other hand, hydroxocobalamin is known to exert a vasopressor effect, leading to an increase in blood pressure.^[9]^ In this case, hypertension persisted from admission without progression to hypotension. This clinical course may indicate that early administration of the antidote mitigated the cardiovascular effects of cyanide toxicity. Alternatively, the persistent hypertension could reflect the pressor effect of hydroxocobalamin.

In addition to clinical conditions, improvements in consciousness and blood circulation, arterial blood ketone ratio, mixed venous oxygen saturation, lactate/pyruvate ratio, and lactate levels are considered useful in determining the effect of treatment in cyanide poisoning.^[10]^ Lactate levels can be rapidly measured using a blood gas analyzer, enabling immediate therapeutic adjustments. In our case, we measured the lactate levels regularly after antidote administration. The patient gradually regained consciousness as lactate levels improved (Fig. 2). We adjusted the treatment approach, such as reducing the oxygen concentration administered and deciding not to administer additional antidotes, based on the effectiveness indicated by the lactate levels.

## 4. Conclusion

In the present case, we used lactate levels as indicators of treatment efficacy in a patient exposed to a lethal dose of cyanide. Lactate levels can be easily measured in any facility, and may be useful in the treatment of cyanide poisoning.

## Acknowledgments

We would like to thank Editage (www.editage.jp) for English language editing.

## Author contributions

**Data curation:** Mitsuko Suzuki, Saki Takeda.

**Formal analysis:** Saki Takeda.

**Investigation:** Mitsuko Suzuki, Saki Takeda.

**Methodology:** Mitsuko Suzuki, Saki Takeda.

**Project administration:** Mitsuko Suzuki.

**Resources:** Mitsuko Suzuki.

**Validation:** Mitsuko Suzuki.

**Visualization:** Mitsuko Suzuki.

**Writing – original draft:** Mitsuko Suzuki, Ken Iseki.

**Writing – review & editing:** Mitsuko Suzuki, Kazuki Sugaya, Masahiro Iwabuchi, Rie Zenda, Tsuyoshi Suzuki, Makoto Onodera, Ken Iseki.
